# Titanium Dioxide Engineered for Near-dispersionless High Terahertz Permittivity and Ultra-low-loss

**DOI:** 10.1038/s41598-017-07019-9

**Published:** 2017-07-26

**Authors:** Chuying Yu, Yang Zeng, Bin Yang, Robert Donnan, Jinbao Huang, Zhaoxian Xiong, Amit Mahajan, Baogui Shi, Haitao Ye, Russell Binions, Nadezda V. Tarakina, Mike J Reece, Haixue Yan

**Affiliations:** 1 School of Engineering and Materials Science, Queen Mary, University of London, London, E1 4NS United Kingdom; 2 School of Electronic Engineering and Computer Science, Queen Mary, University of London, London, E1 4NS United Kingdom; 3 Department of Electronic and Electrical Engineering, University of Chester, Chester, CH2 4NU United Kingdom; 4 College of Materials, Xiamen University, Simen Road, Xiamen, 361005 China; 5 School of Engineering and Applied Science, Aston University, Birmingham, B4 7ET United Kingdom

## Abstract

Realising engineering ceramics to serve as substrate materials in high-performance terahertz(THz) that are low-cost, have low dielectric loss and near-dispersionless broadband, high permittivity, is exceedingly demanding. Such substrates are deployed in, for example, integrated circuits for synthesizing and converting nonplanar and 3D structures into planar forms. The Rutile form of titanium dioxide (TiO_2_) has been widely accepted as commercially economical candidate substrate that meets demands for both low-loss and high permittivities at sub-THz bands. However, the relationship between its mechanisms of dielectric response to the microstructure have never been systematically investigated in order to engineer ultra-low dielectric-loss and high value, dispersionless permittivities. Here we show TiO_2_ THz dielectrics with high permittivity (ca. 102.30) and ultra-low loss (ca. 0.0042). These were prepared by insight gleaned from a broad use of materials characterisation methods to successfully engineer porosities, second phase, crystallography shear-planes and oxygen vacancies during sintering. The dielectric loss achieved here is not only with negligible dispersion over 0.2–0.8 THz, but also has the lowest value measured for known high-permittivity dielectrics. We expect the insight afforded by this study will underpin the development of subwavelength-scale, planar integrated circuits, compact high Q-resonators and broadband, slow-light devices in the THz band.

## Introduction

The Terahertz (THz) band, defined as the electromagnetic frequency domain spanning from 0.1 to 10 THz, has shown attractive applications in materials science^1^, chemistry^2^, communication engineering^3^ and the life sciences^4^. Small-scaled and high-density planar integration are the key to successful development of mass-produced, cost-efficient and light-weight commercial THz products and systems. Substrate-integrated technologies are promising platform for THz-on-chip in the future^5^. However, the vital driving force is a substrate material’s dielectric properties at THz band frequencies, *i.e*. broadband-dispersionless low-loss and high permittivity. Not having these properties is a major constraining factor for developing the next generation of THz devices that will find application in antennas, isolators, modulators, resonators, and also for efficient THz systems capable of operating at gigabit data rates for big data communications^6–8^. Materials with *low dielectric loss* properties translate into THz systems with low insertion loss, yielding an improved signal to noise ratio (S/N), a serviceable system power budget and, ultimately, cost savings. *High permittivity* dielectrics can not only further scale-down the size of packaging in THz systems, but also create a specific dielectric contrast to augment the sub-mm scaled planar integrated THz components, thereby opening possibilities of THz systems that can be planar-integrated and also compatible with small-scaled graphene and other nano-systems^9, 10^. Non-dispersive mechanism in both loss and permittivity properties, can generally supply broad bandwidth operation and is vital to improve data transmission bit-rate in communication systems and also to give greater flexibility in engineering design.

Previous research has involved traditional microwave materials to seek their potential of being applied in THz band. However, linear dielectrics (*e.g*. Al_2_O_3_)^11^, A_5_B_4_O_15_ family (A = Ba, Sr, Mg, Zn; B = Nb, Ta)^12^ and ferroelectrics, like Aurivillius family (*e.g*. SrBi_2_Nb_2_O_9_)^13^, exhibited either low permittivity or high loss in the THz domain. TiO_2_ owns high permittivity (*ca*. 100, in microwave band)^14^, making it a good candidate for THz applications. The state-of-art study on the dielectric properties of TiO_2_ in the THz band showed little progress as the dielectric loss was always high (≥0.01)^11, 15^. The high dielectric loss is related to the complexed defect structure in TiO_2_. TiO_2_ can be easily reduced to oxygen-deficient non-stoichiometric TiO_2−x_ (where x is the oxygen deficiency O_*d*_), at high temperature and/or low oxygen partial pressure^16^. Defect structures, formed due to this reduction, would act as donor centres and contribute to the n-type semiconductor behaviour and high loss^17^.

Here, using spark plasma sintering (SPS) method to minimize the density of trapped porosity (~0.2 *μm*) and defects, we have successfully produced the TiO_2_ ceramics with ultra-low loss of 0.0042 at 0.20 THz, which is the lowest value for known high permittivity dielectrics (*e.g. ε*_*r*_ > 50). Moreover, the low dispersion of the permittivities and losses is negligible in 0.20–0.80 THz band, and this kind of *Broadband and* high performance properties is also a step change in improving the bit-rate in data transmission^18^, compared to that of the popular metamaterial-based components^19^.

## Results

TiO_2_ ceramics were prepared using both conventional sintering (CS) and SPS methods. Samples sintered at 1210, 1250 and 1300 °C using CS method will be referred to as CS1210, CS1250 and CS1300, respectively. A ZnO (1 wt%) sintering aid was used to decrease sintering temperature of the CS samples and increase their density. The SPS samples sintered at 1050, 1200 and 1250 °C will be referred to as SPS1050, SPS1200 and SPS1250, respectively. No sintering aid was added to these materials. The X-Ray diffraction (XRD) data of the CS and SPS ceramics are shown in the Supplementary Figures S1 and S2, respectively. All ceramics are single phase with rutile structure based on XRD data. All the samples had a relative density of greater than 95% (Supplementary Table S1).

**Figure 1:**
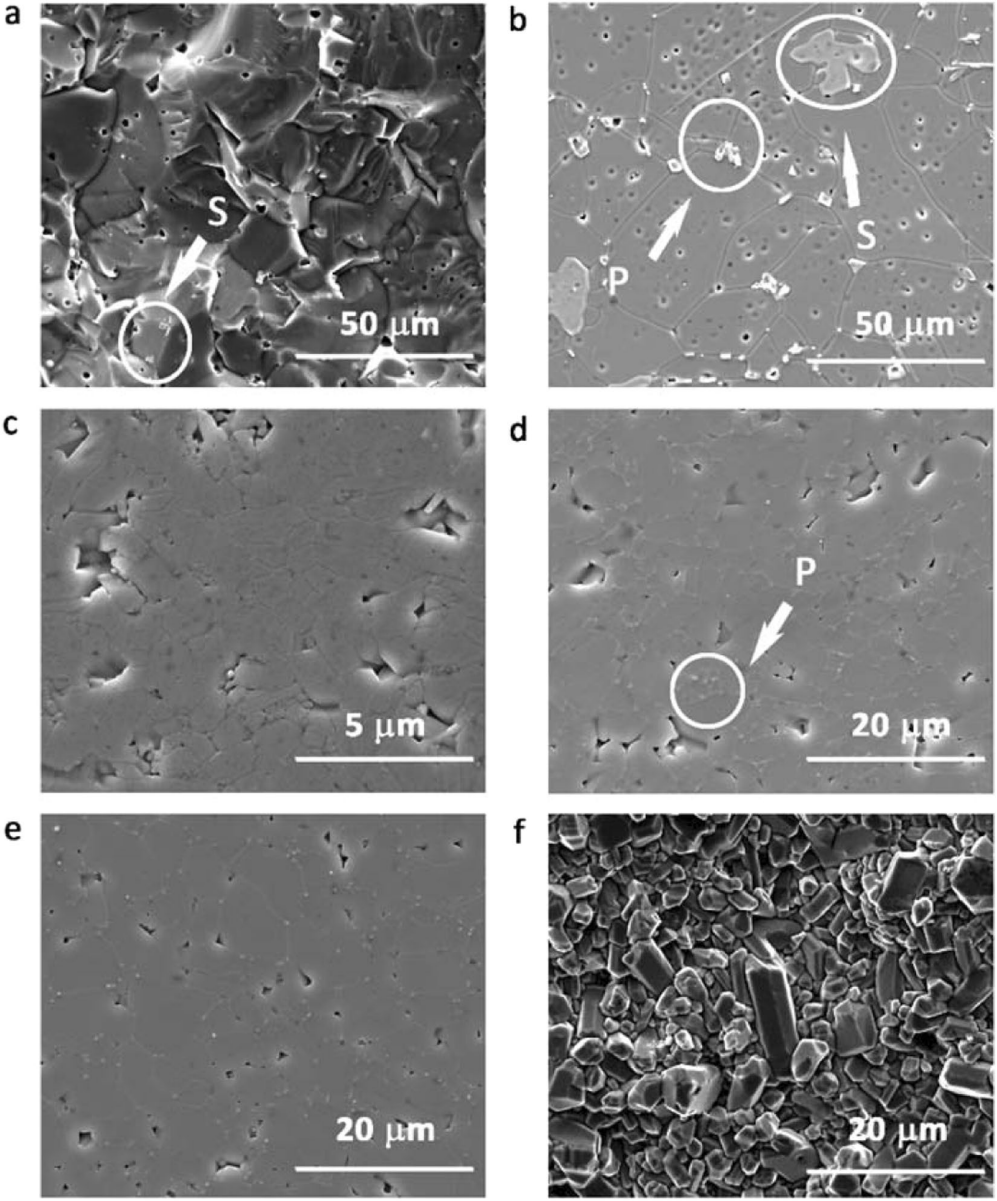
SEM of (**a**) fracture surface of CS1210; (**b**) CS1210 thermally etched at 1150 °C; (**c**) SPS1050 thermally etched at 1000 °C; (**d**) SPS1200 thermally etched at 1050 °C; (**e**) SPS1250 thermally etched at 1100 °C; (**f**) fracture surface of SPS1250 (S denotes sintering aid; P, precipitation).

Figure 1 shows SEM images of the TiO_2_ ceramics. The microstructure of a fracture surface for the CS1210 sample is shown in Fig. 1a. There is a high density of trapped porosities, and sintering aid (marked as S) can be seen as second phase. Figure 1b shows the microstructure of the CS1210 sample thermally etched at 1150 °C. The grain size is quite large (~25 µm). Pores can be seen within the grains and at the grain boundaries. The trapped porosities were produced by abnormal grain growth. The observed precipitates (marked as P), were formed by thermal etching^20, 21^. Porosity, second phases (sintering aid), and interfaces (grain boundary) would increase dielectric loss^22^.

Figure 1c shows the microstructure of the SPS1050 sample. It has a small grain (~1 µm) and pore size (~0.2 µm), and trapped porosity. Figure 1d shows the microstructure of the SPS1200 sample. It has a small and homogeneous grain size (~3 µm). Figure 1e shows the microstructure of the SPS1250 sample. The grain size (~7 µm) is larger than that of the SPS1200 sample. Trapped porosity was barely seen in the SPS1200 and SPS1250 samples, but precipitates were additionally observed. Figure 1f shows the microstructure of a fracture surface of SPS1250. No precipitation was observed, since it only formed by the thermal etching.

**Figure 2:**
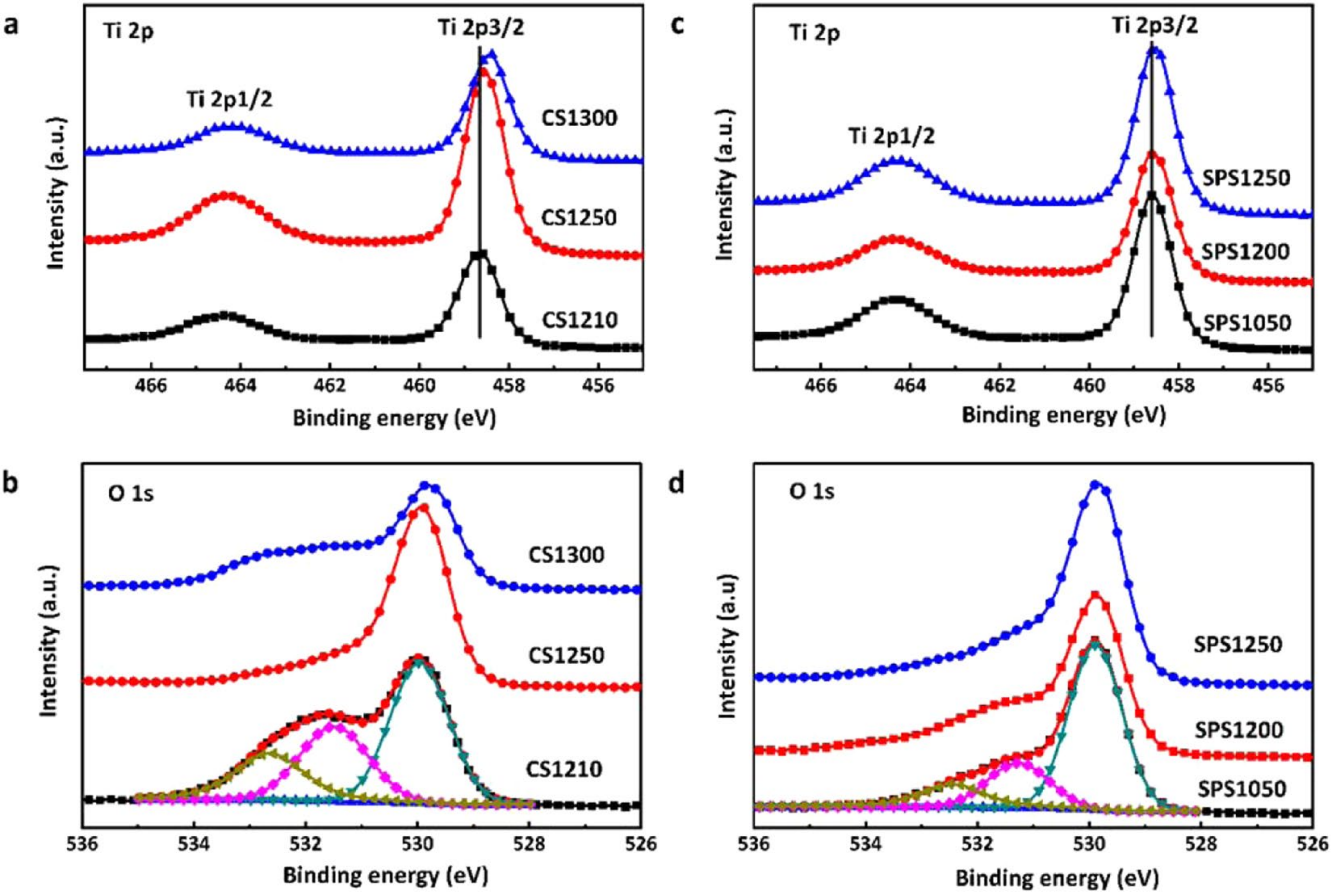
XPS of (**a**) Ti 2p peaks of CS samples; (**b**) O 1 s peaks of CS samples with fitted CS1210; (**c**) Ti 2p peaks of SPS samples; (**d**) O 1 s peaks of SPS samples with fitted SPS1050.

Figure 2 displays the X-ray photoelectron spectroscopy (XPS) characterization of both CS and SPS samples. All the peaks have been fitted using the Lorentzian-Gaussian model^23^. Figure 2a shows the XPS peaks of Ti 2p in CS samples. The Ti 2p3/2 peak shifts slightly towards a lower binding energy with increasing sintering temperature. Figure 2b shows the O 1 s peaks of the CS samples. The O 1 s peak has been fitted with three peaks for each sample and that of CS1210 is shown as an example. The one at 529.98 eV represents oxygen in the intrinsic site; the peak at 531.5 eV is related to oxygen vacancies; and the peak at 532.65 eV is chemisorbed oxygen on the surface of the sample^24^. The amount of oxygen vacancy decreased in CS1250 compared to that in CS1210 and then increased in CS1300. Figure 2c shows the Ti peaks of the SPS samples. The Ti 2p3/2 peak positions of SPS1050 and SPS1200 are the same, but it tends to slightly shifts towards a lower binding energy in SPS1250. Figure 2d shows the O 1 s peaks of the SPS samples, with a fitted example of SPS1050. The amount of oxygen vacancy decreased from SPS1050 to SPS1250.

The shift of Ti 2p3/2 peaks in the CS samples to a lower binding energy is assumed due to the lowering of valence state of a small number of Ti cations from Ti^4+^ to Ti^3+^, which indicates the reduction of TiO_2_ and formation of Ti^3+^ interstitials at high sintering temperature^25^. The intensity of the oxygen vacancy peak increased with increasing sintering temperature, due to the higher thermal activity of oxygen. The formation of oxygen vacancies would lead to non-stoichiometry in the compound^16, 17^. However, a diminishing of oxygen vacancy is observed in CS1250 (Fig. 2b). The formation of crystallographic shear planes in TiO_2_ is suggested to account for the elimination of oxygen vacancy. According to the Anderson and Hyde model^26^, oxygen vacancies would aggregate into disks and then collapse to a shear-plane nucleus bounded by a dislocation loop. The shear plane would grow by trapping vacancies on the loop. Crystal structure then changes from corner-shared octahedrals to edge-shared octahedrals or edge-shared octahedrals to plane-shared octahedrals^26^. It is reported that, in extremely reduced TiO_2_, crystallographic shear planes would exist as an ordering structure with a regular spacing, resulting in a homologous series of stoicheimetrically-defined intermediates with general formula Ti_n_O_2n−1_, also known as Magnéli phase^27^. However, crystallographic shear planes were also observed in pure TiO_2_ ceramics, conventionally sintered in air^28^. Note that, in slightly reduced TiO_2_, crystallographic shear planes could exist in limited nano regions (bounded by dislocation rings) from the clustering of oxygen vacancies, as is in this work^29^.

The formation of crystallographic shear planes in CS1250 are due to the aggregation of oxygen vacancies by a high sintering temperature. However, the shortening of cation-cation distance by crystallographic shear planes leads to an enhancing cation-cation repulsion, which would impede the further growth of crystallographic shear planes^30^. Meanwhile, the formation of oxygen vacancies is temperature-dependent. As the sintering temperature increases, the rate of producing oxygen vacancies outweighs that of eliminating oxygen vacancies by crystallographic shear planes. As a result, the amount of oxygen vacancies increased again in CS1300. The diminishing trace of oxygen vacancy was also observed in SPS1250 (Fig. 2d), suggesting the formation of crystallographic shear planes at high temperature.

**Figure 3:**
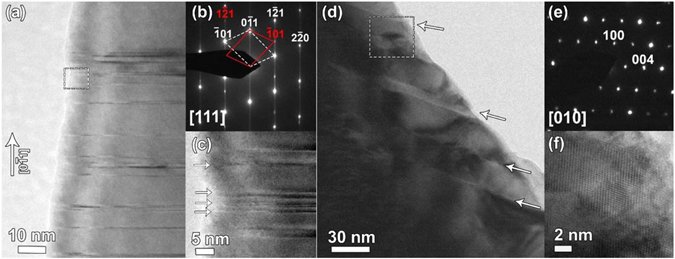
(**a**) HRTEM image of defects in CS1250; (**b**) selected area diffraction pattern (SAED) taken from the crystal in (**a**). Red and white indices indicate reflections on the SAED that are coming from different twin domains in the rutile structure; (**c**) HRTEM image of the area marked by dashed lines in (**a**), showing twin defects; (**d**) bright-field electron microscopy image showing complex defects in CS1250. White arrows indicate positions of faults; (**e**) SAED pattern taken from the area of the crystal with defects shown in (**d**). The pattern is indexed in the Ti_4_O_7_ Magnéli phase. (**f**) HRTEM image of the area marked by dashed lines in (**d**), showing the Ti_4_O_7_ Magnéli phase area of the grain.

Transmission electron microscopy study of the CS1250 revealed the presence of twins and shear planes in the crystals. Figure 3(a–c) show high-resolution transmission electron microscopy (HRTEM) images of (0–11) twins and the corresponding selected area electron diffraction (SAED) pattern. Diffraction patterns of both individual twins can be related by a 180°-rotation about the [0–11] reciprocal lattice vector, or equivalently by a mirror plane operation on the (0–11) plane. Formation of these twins does not change the stoichiometry of either metal or oxygen sublattices. In contrast^29^, the presence of (121) shear planes in rutile leads to the formation of Magnéli phases, with face-shared TiO_6_ octahedra, and an increase in the Ti/O ratio. In case of the CS1250 sample we observe different local ordering of shear planes in the crystals so that areas of oxygen deficiency are formed within crystals. Figure 3(d–f) show an example of HRTEM images and the corresponding SAED pattern of such a local region where the Ti_4_O_7_ structure is formed.

**Figure 4:**
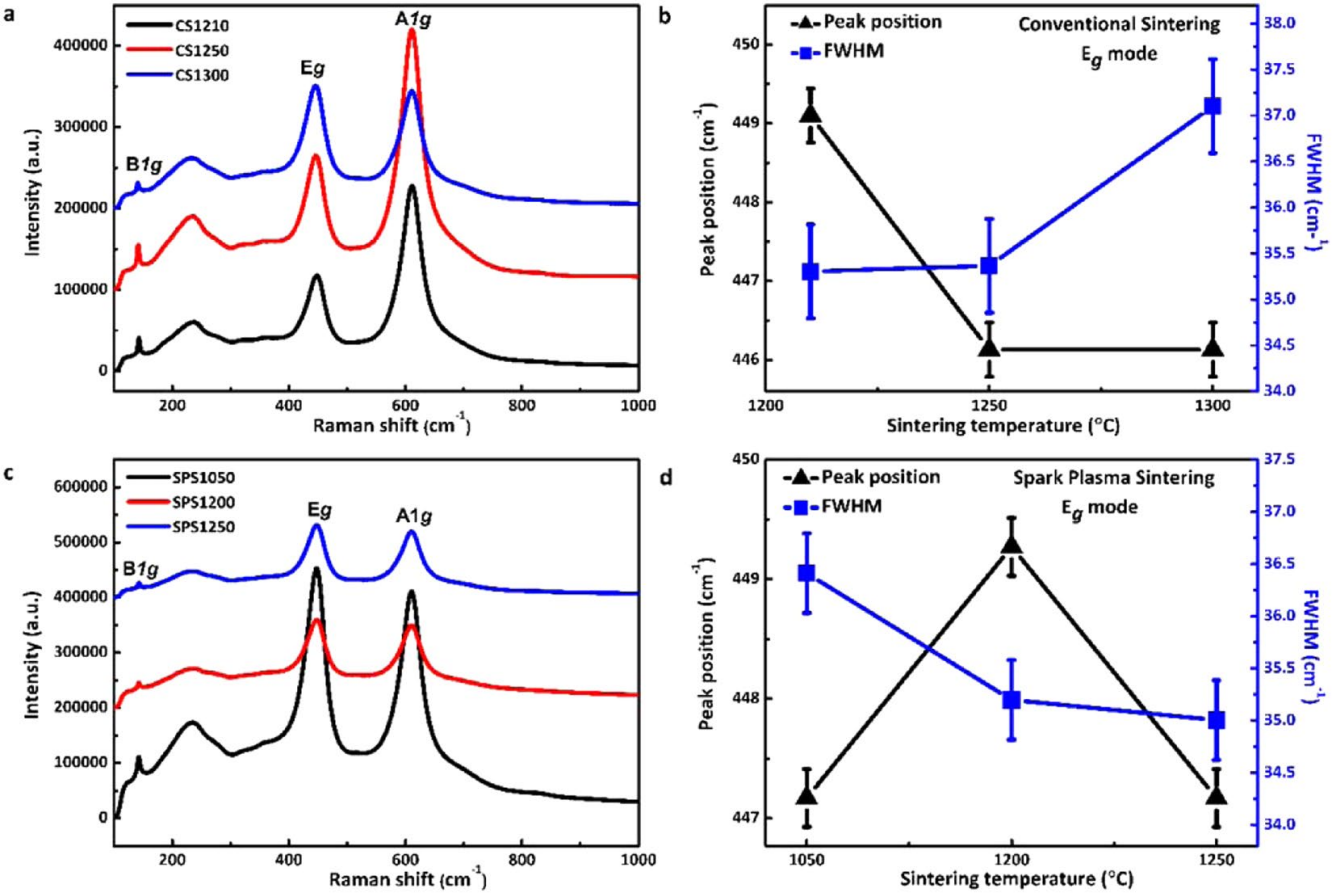
(**a**) Raman spectra of conventional sintering samples; (**b**) peak position and FWHM of the E*g* mode of CS samples; (**c**) Raman spectra of SPS samples; (**d**) peak position and FWHM of E*g* mode of SPS samples.

Figure 4a shows the Raman spectra of CS samples. The peak at *ca*. 142 cm^−1^ is identified as the B*1g* mode, the peak at *ca*. 450 cm^−1^ is the E*g* mode and the peak at *ca*. 608 cm^−1^ is the A*1g* mode. The broad peak at 250 cm^−1^ corresponds to a second order phonon mode^31^. The E*g* mode is associated with a planar O-O vibration mode, while the A*1g* and B*1g* modes are associated with a Ti-O stretching vibration. The oxygen deficiency in these planes would affect the E*g* mode more than the A*1g* and B*1g* modes^31, 32^. Therefore, only the change of the E*g* mode is considered here. The peak position of the E*g* mode was carefully identified and the full width at half maximum (FWHM) was calculated for CS samples (Fig. 4b). The peak position of the E*g* mode is red-shifted and the peak becomes broader as the sintering temperature increases^31^. The red-shift of phonon energies (softening) and the broadening of phonon energies are due to anharmonic behaviour of the crystals. Anharmonicity reduces crystal stability, degrades desired optical and dielectric and thermal transport properties and, causes phonon damping^33, 34^. Phonon softening of the CS samples, with increasing sintering temperature (Fig. 4b), indicated an increase of crystal instability linked with crystallography shear planes. The broadening of phonon energies was caused by the scattering of phonons by defects, which could further increase phonon damping, leading to high dielectric loss.

Raman spectra of the SPS samples are shown in Fig. 4c. The E*g* peak position and FWHM of the SPS samples are shown in Fig. 4d. The softening of the E*g* mode and broadening of peak width in SPS1050, indicated incomplete crystallisation, accompanied by anharmonicity. The peak position blue-shifts from SPS1050 to SPS1200 but it decreases again for SPS1250. The phonon stiffening in SPS1200 demonstrates the increase of crystal stability and the diminishing effect of anharmonicity. SPS1250 experienced a phonon softening due to the appearance of crystallographic shear planes as mentioned in Fig. 2d. The FWHMs keep decreasing as the sintering temperature increases, which could be related to a lowering of dielectric loss.

**Figure 5:**
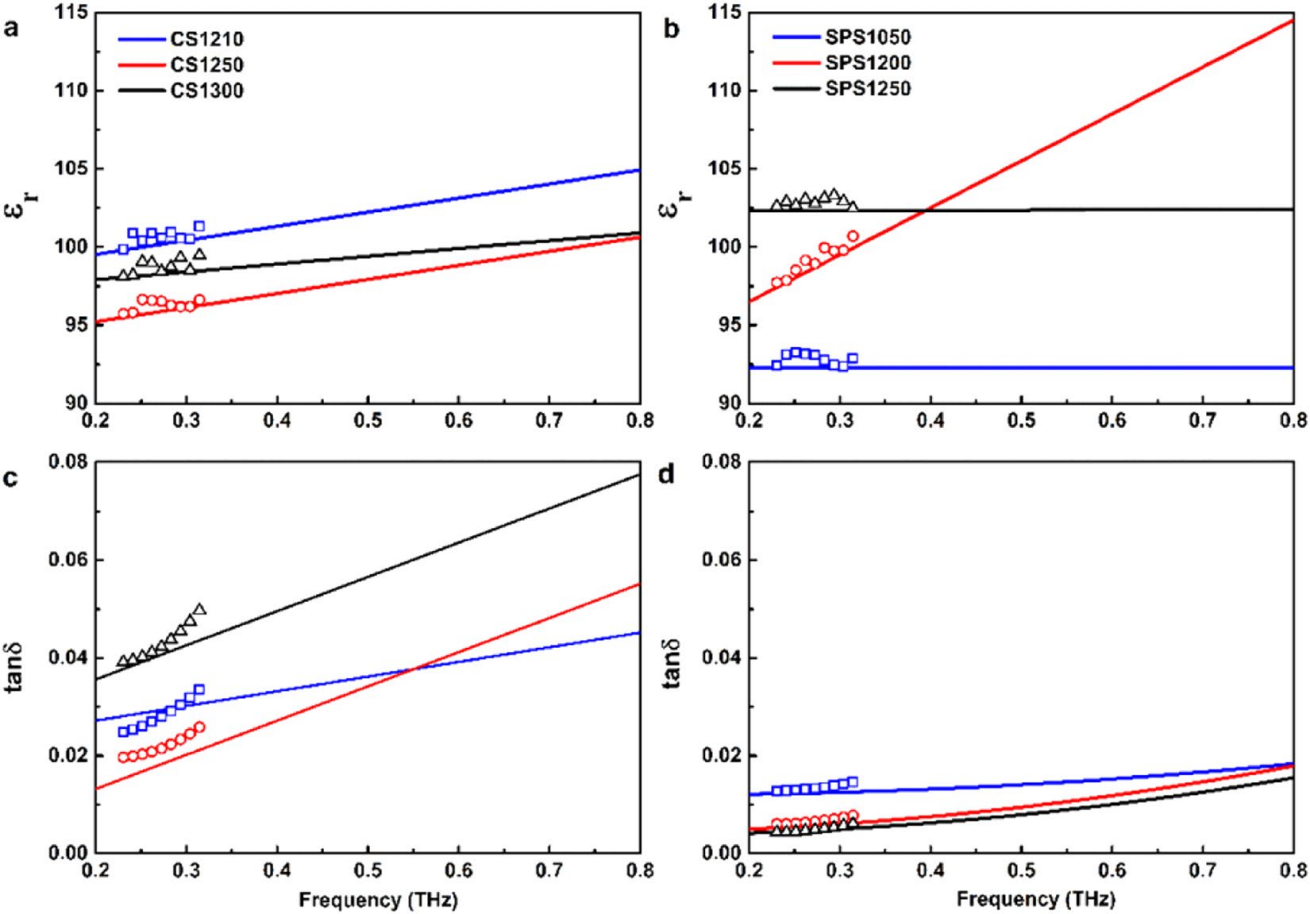
Frequency-dependence of (**a**) permittivity in CS samples; (**b**) permittivity in SPS samples; (**c**) dielectric loss in CS samples and (**d**) dielectric loss in SPS samples in the THz sub-spectral domain (0.20–0.80 THz). The solid lines are the experimental results from THz-TDS system and the discrete points are the experimental results from the VNA + QO transmission system.

Figure 5 shows the frequency dependence of dielectric response of samples in the THz band. High dielectric permittivities (ɛ_r_ > 90) were obtained for all samples. The permittivity of the CS samples decreased from CS1210 to CS1250 and then increased in CS1300 (Fig. 5a). The decrease of permittivity in CS1250 is suggested to be related to the diminishing of oxygen-vacancy-linked dipoles, as discussed in Fig. 2b. Most of the oxygen vacancies would be eliminated by the formation of crystallographic shear planes, and as a result, the contribution from these defect-related dipoles to permittivity would vanish. The growth rate of oxygen vacancies in CS1300 outweighed the formation of crystallography shear planes, and the permittivity of CS1300 increased again due to extra contribution from local vibration of oxygen-vacancy-linked dipoles to the polarizability^35^. For SPS samples (Fig. 5b), dielectric permittivity increased with increasing sintering temperature, which is correlated to the densification of samples and the increase of grain size (Fig. 1). The permittivity of the SPS1200 is dispersive comparing to those of the other samples. It is suggested that the oxygen vacancies started to form clusters in SPS1200. The permittivity of SPS1200 showed clear increase with increasing frequency and become dispersive as it is approaching the harmonic vibration of the cluster under THz radiation with increasing frequency. Whereas, the cluster kept growing with increasing sintering temperature and crystallography shear planes were formed in SPS1250 (Fig. 2d), which is more stable under the tested THz domain. As for SPS1050, the permittivity showed much less dispersive in the tested frequency range as the sintering temperature is too low to form oxygen-vacancy related clusters.

The dielectric loss of the CS samples is generally higher than those of the SPS samples, as seen in Fig. 5c,d. This is associated to the high density of the trapped porosities (Fig. 1) and oxygen vacancies (Figs 2 and 4) existed in CS samples. SPS1050 exhibited trapped porosity and relative low density (Fig. 1c), leading to the high loss. Interest has been focused on SPS1200 and SPS1250 (Fig. 5d), due to the ultra-low loss (0.0050 and 0.0042, respectively) observed, which are less than half of that in SPS1050 (0.0121) and CS1250 (0.0133, and the lowest among the CS samples) at 0.20 THz. Trapped porosity, as a main mechanism for loss, was barely observed in SPS1200 and SPS1250 (Fig. 1). Meanwhile, less anharmonicity from defects has been deduced from the Fig. 4d, which is also taken to account for the low-loss.

The complex dielectric permittivity of SPS1250, fitted by a four-parameter semi-quantum (FPSQ) model, is shown in Figure S3 with comparison to a previous study by Matsumoto *et al*.^36^. The loss tangent has been reduced by 20% at 0.2 THz and 30% at 0.8 THz. The dispersion parameters of SPS1250 extracted from the measured complex transmission data are listed in Table S2 with comparison to the reported data^36^. The red-shift of 1^st^ order TO and LO modes, with significantly decreased damping frequencies, indicates a less dispersive permittivity and lower loss.

**Figure 6:**
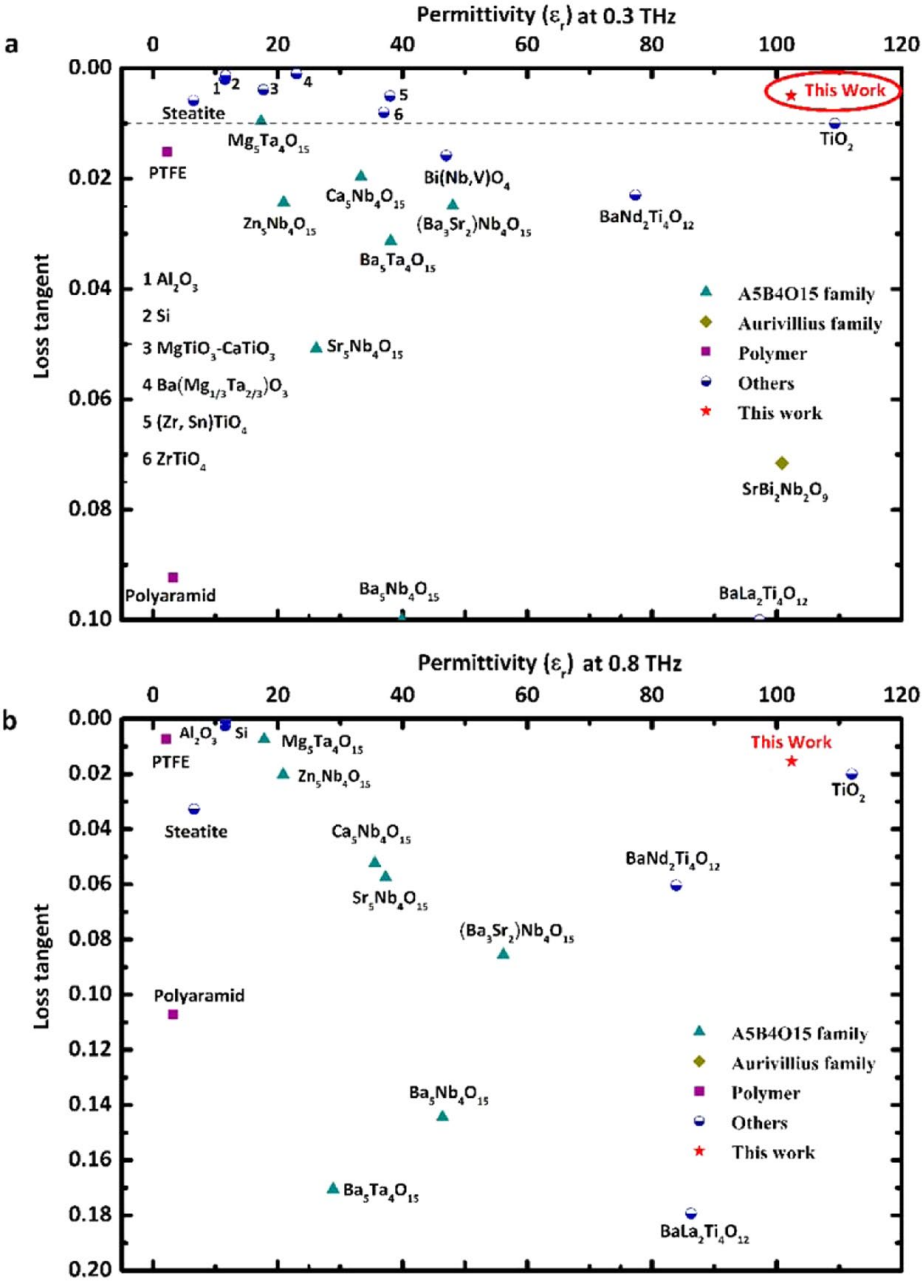
A review of reported dielectric behaviour for different materials at (**a**) 0.3 THz; (**b**) 0.8 THz (references are provided in the supplementary information).

Figure 6 displays the reviewed dielectric behaviour of different materials, including linear dielectrics, ferroelectrics and polymers from the literatures, at both 0.3 and 0.8 THz. The TiO_2_ ceramic produced here has negligible dispersion and ultra-low loss over the tested THz band, which is the lowest value for known high permittivity dielectrics (*e.g. ε*_*r*_ > 50).

In conclusion, we have reproducibly engineered manufactured pure TiO_2_ ceramics with the desired properties of high permittivity and ultra-low loss in the 0.20–0.80 THz band. It has been demonstrated that a low density of trapped porosity, high ceramic density and low concentration of oxygen vacancies are the key factors governing the dielectric properties, which have been achieved using the SPS method of sintering rather than the conventional. Such ceramics are promising for not only devising efficient THz micro-structured components, but also minimizing size and maximizing the resonance quality factor of microelectromechanical and wireless electronic systems.

## Methods

### Sample preparation

The starting materials were TiO_2_ (Alfa Aesar, 99.6%) and ZnO (Sigma-Aldrich, 99.6%). For SPS samples, TiO_2_ ceramics were produced by Spark Plasma Sintering (HPD 25/1 FCT, German), at 1050 °C, 1200 °C and 1250 °C at a pressure of 80 MPa for 3 minutes. The heating and cooling rates were 100 °C min^−1^. Samples were then annealed in air at 100 °C below their sintering temperature for 20 hours to remove any carbon contamination and to equilibrate the oxygen content at atmospheric conditions. For CS samples, TiO_2_ powder mixed with 1 wt% ZnO were sintered at 1210 °C, 1250 °C and 1300 °C for 3 hours in air. Densities were obtained using Archimedes’ method.

### Characterization

The microstructure of the specimens was examined using a scanning electron microscope (FEI, Inspect F, Hillsboro, OR). For the transmission electron microscopy studies the samples were crushed in ethanol. A drop of this dispersion was put on a copper grid covered with a holey carbon film. High-resolution transmission electron microscopy (HRTEM) images and selected area electron diffraction (SAED) patterns were recorded using a JEOL JEM 2010 transmission electron microscope operated at 200 kV. Raman spectroscopy (Renishaw Ramascope, He-Neon Laser-633 nm) was performed on the ceramics. The electron energy states of elements in the samples were determined by X-ray photoelectron spectroscopy (XPS, ESCALAB MK II, VG Scientific). The XPS peaks were fitted using Origin 9.0 software.

### Terahertz Dielectric measurements

The THz complex dielectric response of the samples was measured using two apparatus: one is by standard terahertz time-domain spectroscopy (THz-TDS from TeTechs Ltd) to measure the signal as a function of time-delay between the THz radiation and probe beam, in which the electric field of the THz pulse in the time-domain can be mapped. Fourier transforming the time-domain data yields a spectral domain response workably spanning 0.2 to 0.8 THz for the TiO_2_ samples. The second THz spectrometer is a Vector Network Analyser (VNA, HP N5244A from Keysight Technology Ltd) and its millimetre wave extension heads were used to drive a quasi-optical (QO) transmission meter^37^ to directly acquire frequency scattering parameters from 0.220 to 0.325 THz. Error analysis has been done by measuring sample thickness four times and repeating THz measurement five times. The sample thickness is listed in Table S3.

### Data Availability

All data generated or analysed during this study are included in this published article (and its Supplementary Information files).

## Electronic supplementary material

Supplementary Information
