# Supplementary material for: Titanium Dioxide Engineered for Near-dispersionless High Terahertz Permittivity and Ultra-low-loss
Source: Sci Rep. 2017 Jul 26;7:6639. doi: 10.1038/s41598-017-07019-9 (PMC5529529; doi:10.1038/s41598-017-07019-9)
Supplement: Supplementary file 1 — Supplementary Information [file 41598_2017_7019_MOESM1_ESM.pdf]

Titanium Dioxide Engineered for Near-dispersionless High Terahertz Permittivity and Ultra-low-loss

Chuying Yu[1], Yang Zeng[2], Bin Yang[3,*], Robert S Donnan[2], Jinbao Huang[4], Zhaoxian Xiong[4], Amit Mahajan[1], Baogui Shi[5], Haitao Ye[5], Russell Binions[1], Nadezda V. Tarakina[1], Mike J Reece[1] & Haixue Yan[1,*]

[1] School of Engineering and Materials Science, Queen Mary, University of London, E1 4NS (United Kingdom)

[2] School of Electronic Engineering and Computer Science, Queen Mary, University of London, E1 4NS (United Kingdom)

[3] Department of Electronic and Electrical Engineering, University of Chester, CH2 4NU, (United Kingdom)

[4] College of Materials, Xiamen University, Simen Road, Xiamen, 361005 (China)

[5] School of Engineering and Applied Science, Aston University, Birmingham, B4 7ET (United Kingdom)

Correspondence and requests for materials should be addressed to B.Y. (b.yang@chester.ac.uk) and H.X.Y. (h.x.yan@qmul.ac.uk).

Tables

Table S1 lists the densities of all the samples. The density of the CS samples decreased with increasing temperature to 1300 °C, due to over-sintering. The density of SPS samples increased from SPS1050 to SPS1200 but decreased for SPS1250.

Table S1. Density of samples sintered by both methods (with an error bar ±0.02)

(Theoretical density 4.23 g cm^{-3})

	CS1210	CS1250	CS1300	SPS1050	SPS1200	SPS1250
Density [g cm^{-3}]	4.18	4.17	4.12	4.038	4.17	4.13

Figures

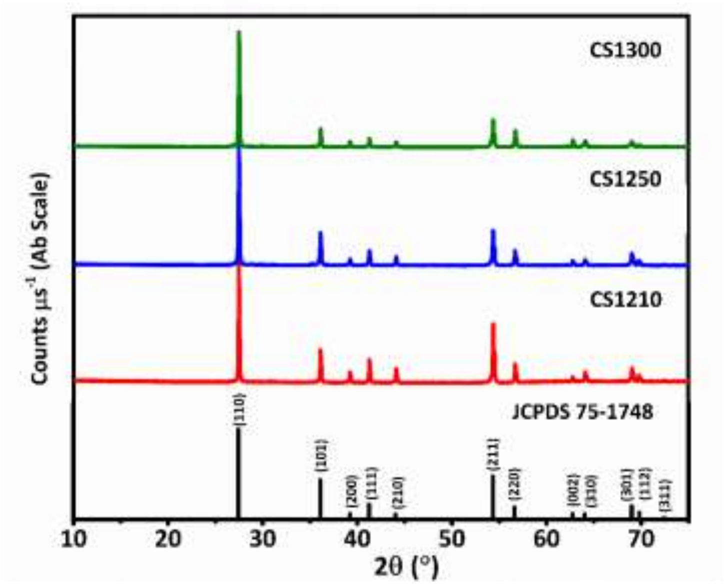

Figure S1. XRD of conventional sintering ceramics (CS1210, CS1250 and CS1300)

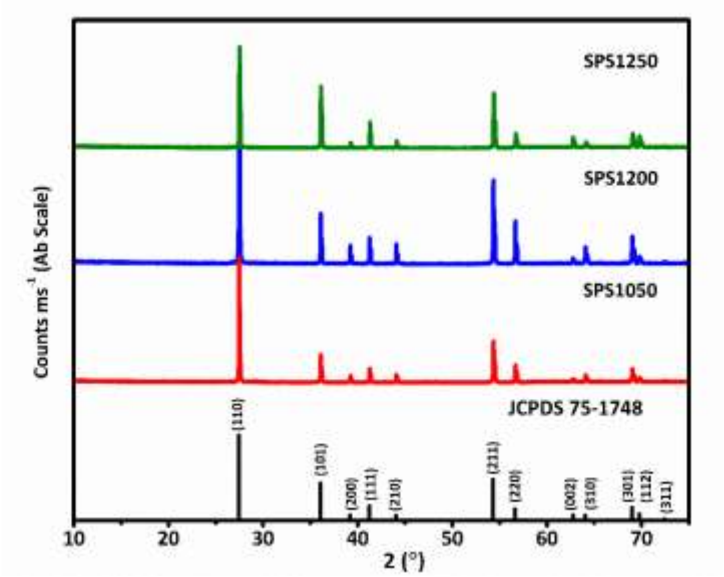

Figure S2. XRD of SPS ceramics (SPS1050, SPS1200 and SPS1250)

Terahertz Data of the TiO_2 samples:

The dielectric parameters of the TiO_2 samples are extracted from the measured complex transmission data based on the algorithms in [1] and [2]. A four-parameter semi-quantum (FPSQ) model is then used for further precise analysis of the dielectric dispersion on sample SPS1250. On the left side of Fig. S3, the measured complex dielectric permittivities from two THz transmissometers are fitted by the FPSQ model. The four parameters are transverse optical (ω_{TO}) and longitudinal optical (ω_{LO}) phonon frequencies and their respective associated damping factors γ_{TO} and γ_{LO}. The subscript 'j' presents the jth transverse and longitudinal optical mode. The detailed parameters are listed in Table S2, and the dispersion parameters reported in Matsumoto's work [3] are also listed in the table and co-plotted in Fig. S3. The fitted four parameters indicate a 1st order red-shift of TO and LO modes with significantly decreased damping frequencies on both modes, which contribute to less dispersion though higher value of permittivity on sample SPS1250. There is also a contribution to lower dielectric loss. Compared with the results in Matsumoto's work the loss tangent has been reduced by 20% at the lower end of 0.2 THz, and nearly 30% at higher end of 0.8 THz.

Table S2: Dielectric dispersion parameters obtained by four-parameter semi-quantum fitting of THz transmission data. The parameters reported in Matsumoto's work [3] are listed in brackets and the work here focused on the 1st mode parameters only.

**Mode (*j*)**	ω_{jTO} (cm^{-1})	γ_{jTO} (cm^{-1})	ω_{jLO} (cm^{-1})	γ_{jLO} (cm^{-1})
**1**	187.5	19	820	1
	(190)	(23)	(821)	(50)
**2**	(379)	(19)	(366)	(9)
**3**	(505)	(19)	(445)	(22)

Error Analysis:

Five measurements on each sample have been performed with care taken to illuminate the same portion of each samples as identically as possible. Furthermore, a standard high resistivity silicon wafer (3,000 Ωcm and thickness 1.046 ± 0.001 mm), is measured as standard reference. The thicknesses of TiO_2 samples are measured four times and the average values are listed in Table S3. The maximum thickness uncertainty of ±9 µm propagates to give a maximum error in ε' of 0.5% and in ε'' of 2%, for VNA-based frequency-domain analysis and, an error in ε' of 1% and ε'' of 5% for THz time-domain based spectroscopy. The calculation method, uncertainty analysis and comparison between these two systems have been reported in Sun's work [4]. On the right-handed side of Fig. S3, the complex permittivities of sample SPS1250 in the domain of 0.2 to 0.8 THz have been magnified while showing associated error bars. Simultaneous achievement of high permittivity and low loss properties has been significantly advanced, and clearly characterised by application of FPSQ model analysis.

Table S3: Thickness Measurement (mm)

Sample	1st	2nd	3rd	4th	Mean	Uncertainty
**SPS1050**	2.559	2.562	2.578	2.558	2.564	±0.009
**SPS1200**	3.141	3.155	3.164	3.152	3.153	±0.009
**SPS1250**	3.358	3.360	3.358	3.352	3.357	±0.003
**CS1210**	0.992	0.996	0.992	0.990	0.993	±0.003
**CS1250**	0.991	0.990	0.994	0.991	0.992	±0.002
**CS1300**	1.040	1.052	1.035	1.039	1.042	±0.007

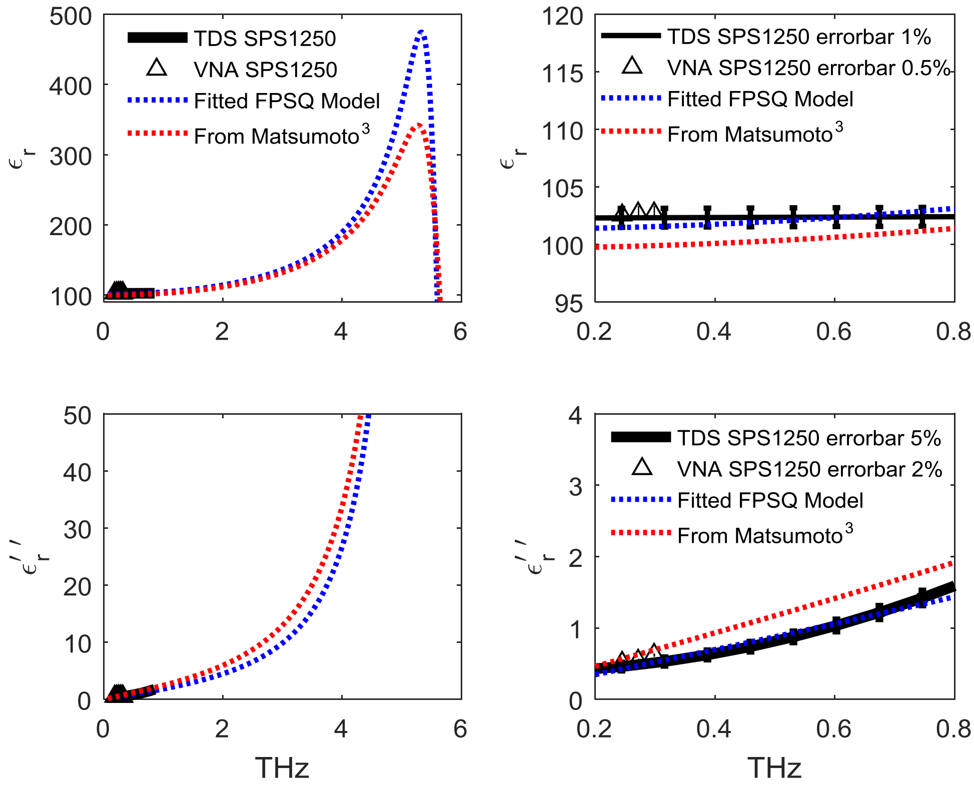

Figure S3: Complex permittivities of the sample SPS1250 with FPSQ model fitting. The dispersive dielectric parameters reported in Matsumoto's work [3] are also co-plotted here.

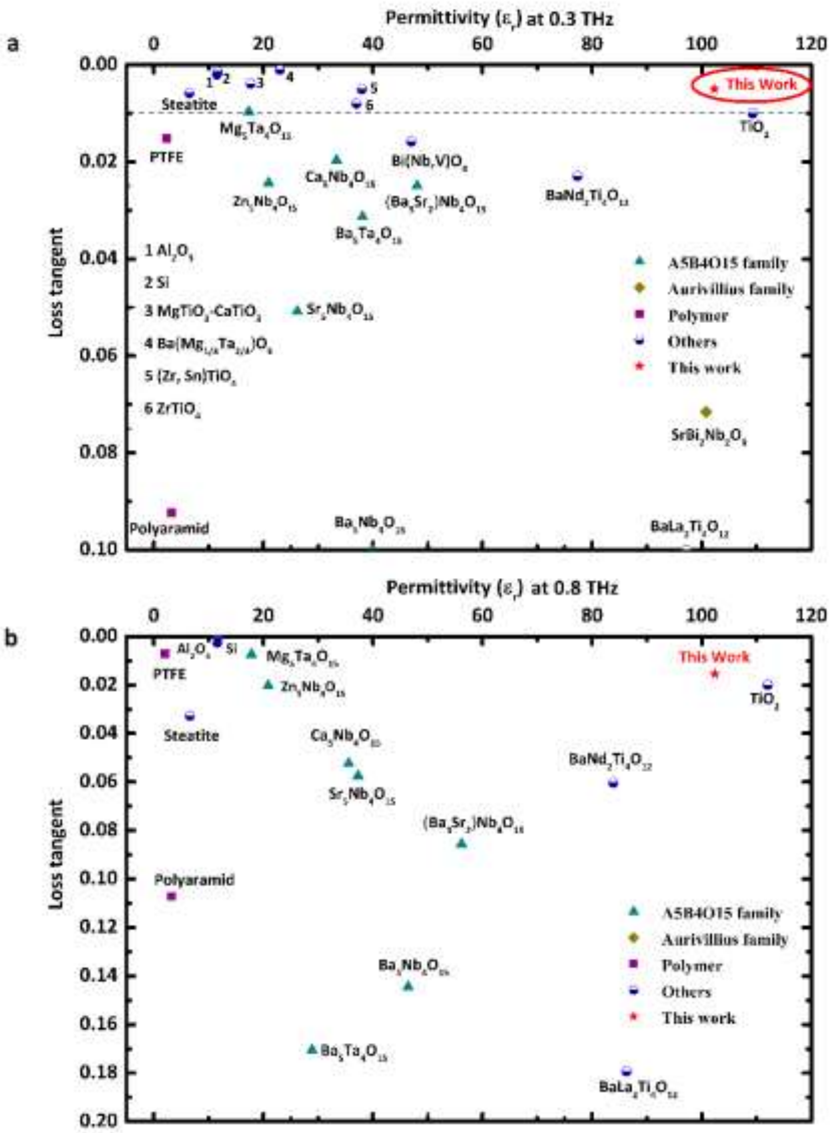

Figure 5: A review of reported dielectric behaviour for different materials at (a) 0.3 THz; (b) 0.8 THz.

Materials include: $A_5B_4O_{15}$ family ($Ba_5Nb_4O_{15}$, $(Ba_3Sr_2)Nb_4O_{15}$, $Ba_5Ta_4O_{15}$, $Ca_5Nb_4O_{15}$, $Mg_5Ta_4O_{15}$, $Sr_5Nb_4O_{15}$, $Zn_5Nb_4O_{15}$)[5]; Aurivillius family ($SrBi_2Nb_2O_9$)[6]; polymers (poly tetrafluoroethylene (PTFE), polyaramid)[7]; and some other materials (Al_2O_3[8], TiO_2[8], $BaNd_2Ti_4O_{12}$[9], $BaLa_2Ti_4O_{12}$[9], $Ba(Mg_{1/3}Ta_{2/3})O_3$[9], Bi(Nb, V)O_4[10], $MgTiO_3$-$CaTiO_3$[11], Si[12], $ZrTiO_4$[9], (Zr, Sn)TiO_4[9], steatite[12])

Reference

1 Yang, B. *et al.* Determination of the gyrotropic characteristics of hexaferrite ceramics from 75 to 600 GHz. *IEEE Trans. Microwave Theory Tech.* **58**, 3587-3597 (2010).

2 Duvillaret, L., Garet, F. & Coutaz, J.-L. A reliable method for extraction of material parameters in terahertz time-domain spectroscopy. *IEEE J. Sel. Top. Quantum Electron.* **2**, 739-746 (1996).

3 Matsumoto, N. *et al.* Analysis of dielectric response of TiO_2 in terahertz frequency region by general harmonic oscillator model. *Jpn. J. Appl. Phys.* **47**, 7725 (2008).

4 Sun, W., Yang, B., Wang, X., Zhang, Y. & Donnan, R. Accurate determination of terahertz optical constants by vector network analyzer of Fabry–Perot response. *Opt. Lett.* **38**, 5438-5441 (2013).

5 Kamba, S. *et al.* High frequency dielectric properties of $A_5B_4O_{15}$ microwave ceramics. *J. Appl. Phys.* **89**, 3900-3906 (2001).

6 Nuzhnyy, D. *et al.* Dynamics of the phase transitions in Bi-layered ferroelectrics with Aurivillius structure: Dielectric response in the terahertz spectral range. *Phys. Rev. B* **74**, 134105 (2006).

7 Jin, Y.-S., Kim, G.-J. & Jeon, S.-G. Terahertz dielectric properties of polymers. *J. Korean Phys. Soc.* **49**, 513-517 (2006).

8 Matsumoto, N., Nakagawa, T., Kageyama, K., Wada, N. & Sakabe, Y. Terahertz Band-Pass filter fabricated by multilayer ceramic technology. *Jpn. J. Appl. Phys.* **45**, 7499 (2006).

9 Petzelt, J., Kamba, S., Kozlov, G. & Volkov, A. Dielectric properties of microwave ceramics investigated by infrared and submillimetre spectroscopy. *Ferroelectr.* **176**, 145-165 (1996).

10 Kamba, S. *et al.* Correlation between infrared, THz and microwave dielectric properties of vanadium doped antiferroelectric BiNbO 4. *J. Eur. Ceram. Soc.* **26**, 2861-2865 (2006).

11 Huang, J. *et al.* Microwave and terahertz dielectric properties of $MgTiO_3$–$CaTiO_3$ ceramics. *Mater. Lett.* **138**, 225-227 (2015).

12 Bolivar, P. H. *et al.* Measurement of the dielectric constant and loss tangent of high dielectric-constant materials at terahertz frequencies. *IEEE Trans. Microwave Theory Tech.* **51**, 1062-1066 (2003).
